# Light regulation of the biosynthesis of phenolics, terpenoids, and alkaloids in plants

**DOI:** 10.1038/s42003-023-05435-4

**Published:** 2023-10-18

**Authors:** Yongliang Liu, Sanjay K. Singh, Sitakanta Pattanaik, Hongxia Wang, Ling Yuan

**Affiliations:** 1 Department of Plant and Soil Sciences and Kentucky Tobacco Research and Development Center, University of Kentucky, Lexington, KY 40546 USA; 2 Shanghai Chenshan Plant Science Research Center, Chinese Academy of Sciences Chenshan Botanical Garden, 3888 Chenhua Road, 201602 Songjiang, Shanghai China

**Keywords:** Secondary metabolism, Light responses

## Abstract

Biosynthesis of specialized metabolites (SM), including phenolics, terpenoids, and alkaloids, is stimulated by many environmental factors including light. In recent years, significant progress has been made in understanding the regulatory mechanisms involved in light-stimulated SM biosynthesis at the transcriptional, posttranscriptional, and posttranslational levels of regulation. While several excellent recent reviews have primarily focused on the impacts of general environmental factors, including light, on biosynthesis of an individual class of SM, here we highlight the regulation of three major SM biosynthesis pathways by light-responsive gene expression, microRNA regulation, and posttranslational modification of regulatory proteins. In addition, we present our future perspectives on this topic.

## Abstract (web summary)

This review highlights the regulation of three major specialized metabolite biosynthesis pathways in plants by light-responsive gene expression, microRNA regulation, and posttranslational modification of regulatory proteins.

## Introduction

Light is a crucial environmental factor that significantly impacts the growth, development, and metabolism in plants. It serves as the primary energy source for photosynthesis while acting as a complex signaling input that modulates plant physiology and development^1^. The perception of light signal and the molecular mechanism by which light regulates developmental and metabolic pathways have been extensively studied in the model plant Arabidopsis. Plants use different photoreceptors to perceive various wavelengths of light. The major photoreceptors include phytochromes, cryptochromes, and the UV Resistance Locus 8 (UVR8). Phytochromes (PHYA-PHYE) perceive red and far-red light, cryptochromes (CRY1 and CRY2) sense blue/UV-A light, and UVR8 detects UV-B light^1–6^. Upon perception of different wavelengths of light, these photoreceptors become activated and transmit signals to downstream components, which consist of transcriptional activators and repressors that regulate diverse light-induced biological processes. The most well-characterized light signaling components include the Phytochrome Interacting Factors (PIFs), Elongated Hypocotyl 5 (HY5), the B-box proteins (BBX), Constitutive Photomorphogenic 1 (COP1), Suppressor of PHYA-105 (SPA), and De-Etiolated 1(DET1)^3–5,7–9^. PIFs are a group of basic helix-loop-helix (bHLH) transcription factors (TFs) that accumulate in the dark and act as repressors of light signaling by inhibiting the expression of light-responsive genes. However, under light conditions, the activated phytochromes directly interact with PIFs, leading to the phosphorylation, ubiquitination, and proteasomal degradation of PIFs^4,5^. Like phytochromes, cryptochromes directly interact with PIFs to regulate their activities on target genes^10^. HY5, a basic leucine-zipper (bZIP) TF, is an activator of different light responses mediated by phytochromes, cryptochromes, and UVR8. HY5 acts synergistically or antagonistically with PIFs to regulate the expression of light-responsive genes in developmental and metabolic pathways in Arabidopsis^1,8^. For instance, HY5 and PIF3 collaboratively regulate anthocyanin biosynthesis^11^, whereas HY5 and PIF1 act antagonistically to regulate carotenoid and chlorophyll pathway genes^12^.

The stabilities of PIFs and HY5 are regulated by COP1/SPA and DET1, which are components of the E3 ubiquitin ligase complexes^13^. COP1, SPA, and DET1 are repressors of light-mediated responses in plants^7,13,14^. The E3 ligase activity of COP1 depends on its interaction with SPA^13^. In the dark, active COP1/SPA and DET1 contribute to the ubiquitination and degradation of HY5, while promoting the stability of PIFs^13^. However, in the presence of light, the active photoreceptors suppress COP1/SPA and DET1 activity, resulting in the stabilization of HY5 and degradation of PIFs^13,15,16^. A recent study suggests that DET1 physically interacts with COP1. DET1 mediates the COP1-HY5 interaction and is necessary for the destabilization of COP1 in a light-independent manner. DET1-mediated COP1 destabilization is necessary for HY5 degradation^13^. The BBX proteins belong to a subfamily of zinc finger TFs with one or two B-box domains at the N-termini. BBX proteins play diverse roles in regulating light signaling through interactions with HY5 and PIFs^17^. In addition to HY5, both COP1 and DET1 are involved in targeting BBX proteins for degradation in darkness^18,19^.

**Fig. 1:**
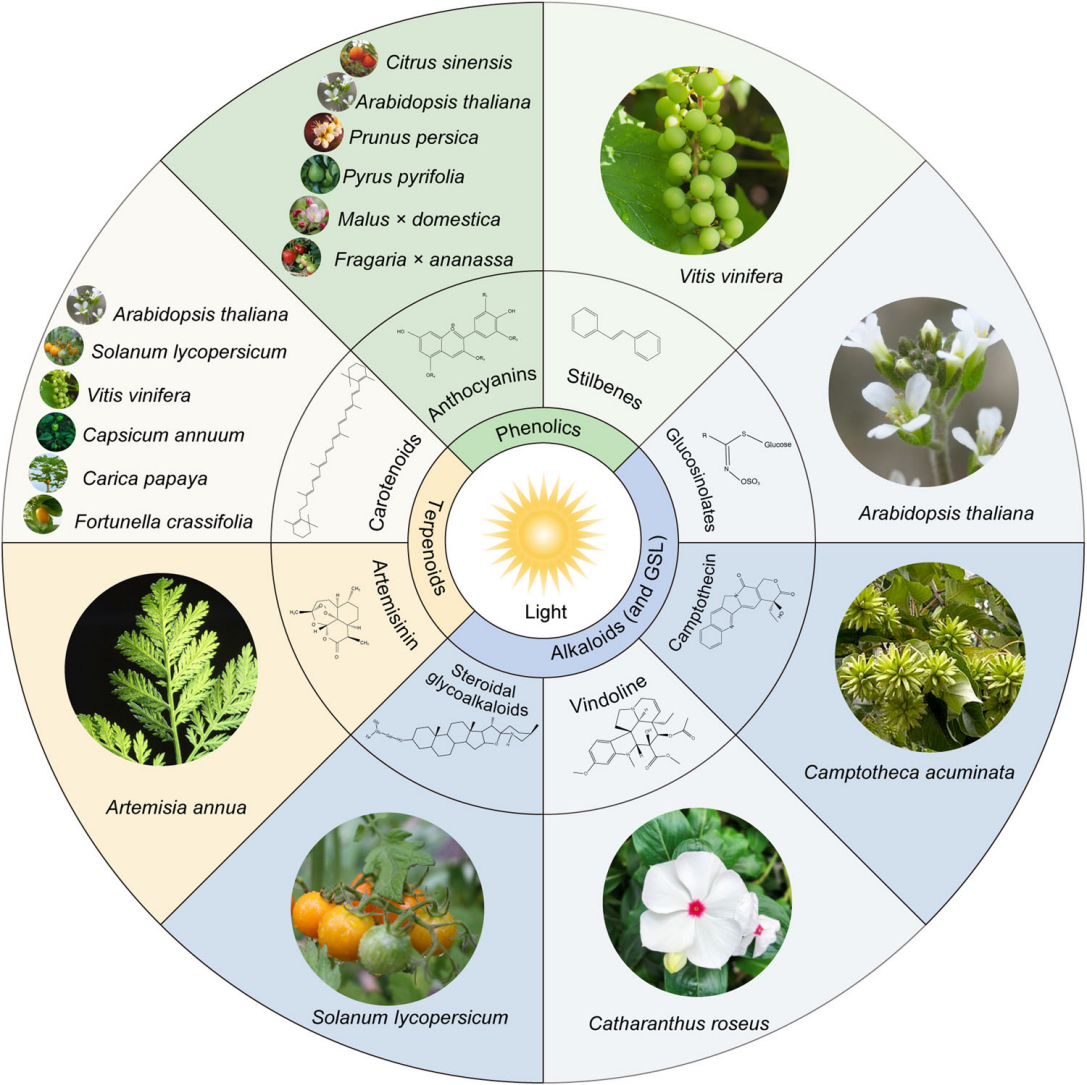
Light regulation of the biosynthesis of plant specialized metabolites (SM). Light affects the biosynthesis of phenolics (anthocyanins and stilbenes), terpenes (carotenoids and artemisinin), alkaloids, and other nitrogen-containing compounds (vindoline, camptothecin, steroidal glycoalkaloids, and glucosinolates) in plants. Certain SM, such as carotenoids and anthocyanins, are produced in a wide range of plants, while others, such as vindoline and camptothecin, are species-specific. The representative plant species include *Arabidopsis thaliana* (Arabidopsis), *Artemisia annua* (Sweet Wormwood), *Carica papaya* (Papaya), *Citrus sinensis* (Citrus), *Camptotheca acuminata* (Chinese Happy Tree), *Capsicum annuum* (Pepper), *Catharanthus roseus* (Madagascar Periwinkle), *Fragaria ananassa* (Strawberry), *Fortunella crassifolia* (Sweet Kumquat), *Malus* x *domestica* (Apple), *Pyrus pyrifolia* (Pear), *Prunus persica* (Peach), *Solanum lycopersicum* (Tomato), and *Vitis vinifera* (Grape).

Plants synthesize more than 200,000 chemically diverse specialized metabolites (SM)^20^. Many SM serve as chemical defenses to protect plants from various biotic and abiotic stresses. In addition, many of these SMs are valued for their inherent therapeutic properties that are beneficial to human health. Light plays a pivotal role in the biosynthesis of an array of SM. Several excellent reviews have covered effects of light on biosynthesis of an individual class of SM such as anthocyanins^21,22^. Zhang et al.^21^ summarize the effects of light quality and intensity on gene expression and metabolite accumulation, while Hashim et al^22^ highlight the use of artificial light sources to elicit SM production in in vitro cultured medicinal plants. However, these reviews have not delved into the underlying regulatory mechanisms that govern SM biosynthesis in response to light. Two recent reviews have included brief summaries of light regulation of anthocyanin biosynthesis in plants^23,24^. In this review, we delve into the light regulation of three major groups of SM: phenolic compounds (e.g., anthocyanins and stilbenes), terpenes (e.g., carotenoids and artemisinin), and alkaloids and nitrogen-containing metabolites (e.g., terpenoid indole alkaloids; steroidal glycoalkaloids and glucosinolates) (Fig. 1). Transcriptional regulation has emerged as the major mechanism governing the biosynthesis of SM^25^. Here, we summarize recent advancements in understanding the transcriptional regulation of selected SM influenced by light. In addition to transcriptional regulation, fine-tuning the accumulation of SM in plants involves post-transcriptional (e.g., microRNA/small RNA)^26–29^ and post-translational (e.g., protein phosphorylation and ubiquitination)^30–32^ regulatory mechanisms. We thus aim to highlight the influence of light on post-transcriptional and post-translational regulation of SM, with a particular emphasis on phenolic compounds such as anthocyanin. In addition, we discuss the future prospect and emerging areas of SM biosynthesis in plants.

### Phenolic compounds: transcriptional, post-transcriptional, and post-translational regulation of anthocyanin biosynthesis in response to light

**Fig. 2:**
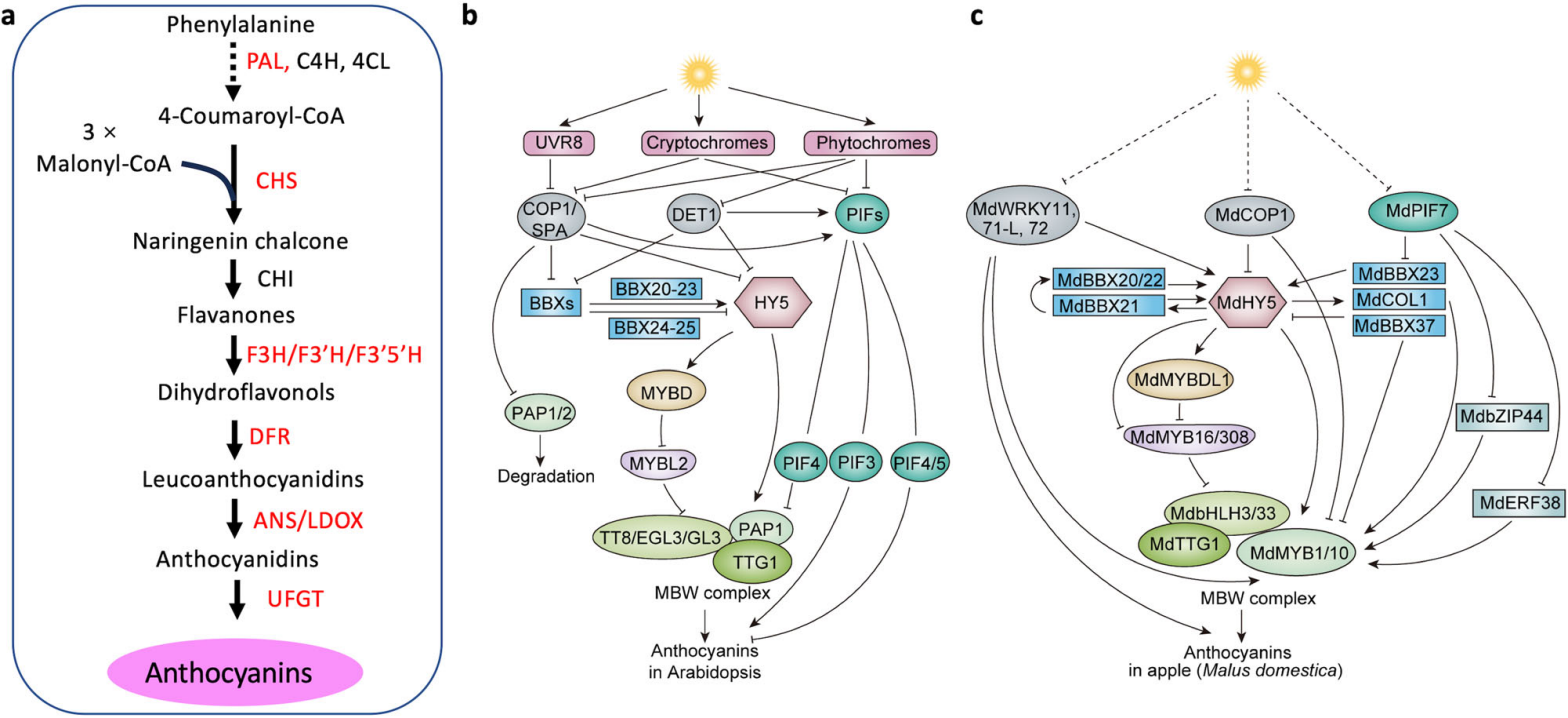
Transcriptional regulation of anthocyanin biosynthesis in Arabidopsis and apple in response to light. **a** Schematic diagram of the anthocyanin biosynthetic pathway in plants. The light-responsive genes encoding anthocyanin biosynthetic pathway enzymes are highlighted *red*. PAL phenylalanine ammonia-lyase, C4H cinnamate 4-hydroxylase, 4CL 4-coumarate: CoA ligase, CHS chalcone synthase, CHI chalcone isomerase, F3H flavanone 3-hydroxylase, F3′H flavonoid 3′-hydroxylase, F3′5′H flavonoid 3′,5′-hydroxylase, DFR dihydroflavonol reductase, ANS/LDOX anthocyanin synthase (leucoanthocyanidin dioxygenase), UFGT UDP-glucose: flavonoid 3-O-glucosyltransferase. The regulations of anthocyanins in Arabidopsis (**b**) and apple (**c**) share significant regulatory mechanisms that are conferred by conserved transcription factors; however, additional characterized regulatory factors are only found in apples. In Arabidopsis and apple (*Malus domestica*), anthocyanin biosynthesis is regulated by transcriptional activators such HY5, BBXs, and the MYB-bHLH-WD40 (MBW) complex, as well as the repressors COP1, DET1, and PIFs. These regulatory factors act downstream of the photoreceptors (UVR8, Phytochromes, and Cryptochromes). HY5 plays a central role in the regulation by activating the expression of the MYB factors (PAP1 in Arabidopsis and MdMYB1/10 in apple) in the MBW complex. HY5 also regulates the expression of the bHLH factors (TT8/EGL3/GL3 in Arabidopsis and MdbHLH3/33 in apple) in the MBW complex through two other MYBs. BBXs differentially regulate HY5 activity to modulate anthocyanin biosynthesis. PIFs act as activators or repressors in the process. PIFs regulate anthocyanin biosynthesis either by targeting PAP1 or the pathway genes. In apple, two additional activators, MdbZIP44 and MdERF38, are involved in MdPIF7-mediated anthocyanin biosynthesis. Furthermore, three apple WRKYs are involved in the regulation of anthocyanin biosynthesis by activating HY5, MdMYB1/10 or pathway genes. bZIP44 BASIC LEUCINE ZIPPER 44, BBX B-box proteins, COP1 CONSTITUTIVE PHOTOMORPHOGENIC 1, DET1 DE-ETIOLATED 1, EGL3 ENHANCER OF GLABRA 3, GL3 GLABRA 3, HY5 ELONGATED HYPOCOTYL5, ERF38 ETHYLENE RESPONSE FACTOR 38, PAP1 PRODUCTION OF ANTHOCYANIN PIGMENT 1, PIFs PHYTOCHROME INTERACTING FACTORs, TT8 TRANSPARENT TESTA 8, TTG1 TRANSPARENT TESTA GLABRA 1, UVR8 UV-RESISTANCE LOCUS8. Solid arrows indicate direct regulation, and the dotted arrows refer to indirect regulation. Solid T-bars and dotted T-bars indicate direct and indirect repression, respectively.

**Fig. 3:**
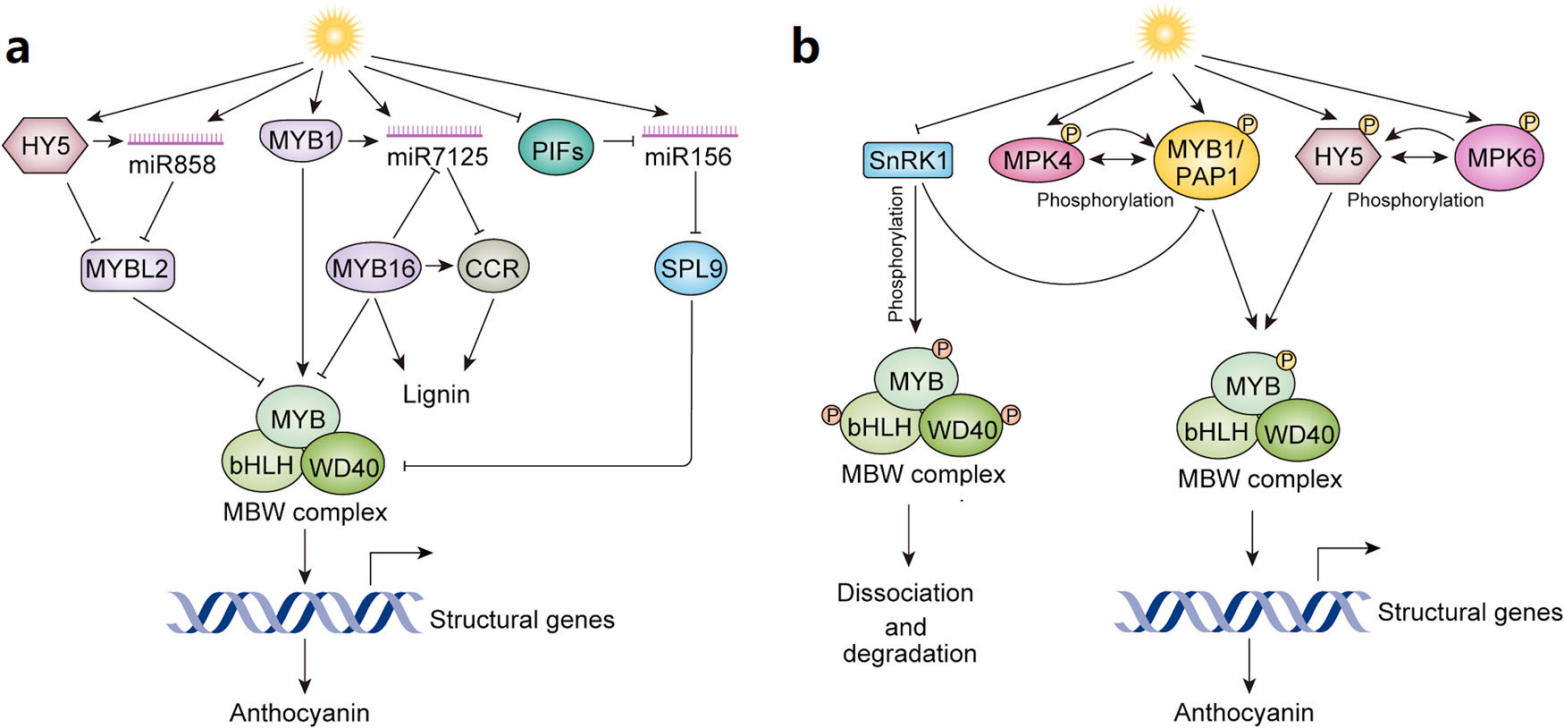
Posttranscriptional and posttranslational regulation of specialized metabolite biosynthesis in response to light. **a** Light and miRNA-mediated regulatory network of anthocyanin biosynthesis. Light induces the expression of several transcription factors (including HY5, MYB1, and PIFs) and miRNAs (miR858, miR7125, and miR156). The expression of miRNAs is differentially regulated by HY5, MYB1, and PIFs. MYB1 positively regulates the MBW complex, while HY5 represses the MBW repressor MYBL2. The light-inducible miR156 positively regulates MBW by repressing the repressor SPL9. MYB16, an activator of lignin pathway gene *CCR*, also represses MBW, thus maintaining the homeostasis between anthocyanin and lignin in response to light. **b** Light and protein kinase-mediated regulatory network of anthocyanin biosynthesis. Light induces the expression of *MAP kinases* (*MPK4* and *MPK6*) which phosphorylate HY5, as well as PAP and MYB1, positively regulating anthocyanin biosynthesis. The kinase SnRK1, a negative regulator of the MBW complex, is suppressed by light. Phosphorylation of the components in the MBW complex leads to its dissociation and degradation, resulting in reduced anthocyanin accumulation. CCR CINNAMOYL-CoA REDUCTASE. MPK4 Mitogen-Activated Protein Kinase 4, MPK6 Mitogen-Activated Protein Kinase 6, PAP1 PRODUCTION OF ANTHOCYANIN PIGMENT 1, PIFs PHYTOCHROME INTERACTING FACTORs, SnRK1 SNF1-related protein kinase 1, SPL9 SQUAMOSA PROMOTER BINDING PROTEIN-LIKE9, CCR Cinnamoyl-CoA reductase, WD40,TRANSPARENT TESTA GLABROUS1. The arrows and T-bars represent positive and negative regulation, respectively. The double-headed arrows indicate the physical interactions between kinases and their target proteins. The phosphate moieties (P) with a positive effect on regulation are depicted in yellow while those with a negative effect are in red.

Anthocyanins are a diverse group of phenolic compounds that serve various biological functions and are accumulated in abundance in flowers, fruits, and vegetables (Fig. 1). Anthocyanins play crucial roles in plant growth, development, and reproduction, and they also provide health benefits to humans^33^. The biosynthesis of anthocyanins has been extensively studied across a wide range of plant species. Anthocyanins are synthesized through the phenylpropanoid pathway in which phenylalanine acts as the primary precursor for all flavonoids, including anthocyanins (Fig. 2a). Phenylalanine undergoes a series of enzymatic reactions to produce coumaroyl-CoA. One coumaroyl-CoA molecule condenses with three malonyl Co-A molecules, catalyzed by chalcone synthase (CHS), to produce naringenin chalcone. Subsequently, chalcone isomerase (CHI) isomerizes naringenin chalcone to form flavanones. Flavanones are then converted to dihydroflavonols by flavanone 3-hydroxylase (F3H) and further reduced to leucoanthocyanidins by dihydroflavonol reductase (DFR). The leucoanthocyanidins are converted to anthocyanidins by leucoanthocyanidin dioxygenase/anthocyanidin synthase (LDOX/ANS)^34^. The expression of anthocyanin biosynthetic genes is primarily regulated by the MYB-bHLH-WD40 (MBW) TF complex, formed by the R2R3MYB, bHLH, and WD40 proteins. In Arabidopsis, the well-studied members of the MBW complex include the R2R3MYBs, Production of Anthocyanin Pigment 1/2 (PAP1/PAP2), the bHLH TFs, Glabrous 3/Enhancer of Glabrous 3/ Transparent Testa 8 (GL3/EGL3/TT8), and the WD40 protein, Transparent Testa Glabra1 (TTG1)^24,35,36^. TF families of AP2/ERFs (Apetala2/Ethylene Response Factors), WRKY, bZIP, and R3MYB have also been identified as regulators of anthocyanin biosynthesis^23,24,35,36^. Light influences anthocyanin biosynthesis through regulating the individual TFs in the MBW complex or the associated upstream TFs. Key TFs involved in the light signaling pathway, such as HY5, BBX, and PIFs, are the major regulators of anthocyanin biosynthesis as they affect the MBW complex. Here, we discuss transcriptional, post-transcriptional (miRNA), and post-translational (phosphorylation/ubiquitination) regulation of anthocyanin biosynthesis under the influence of light (Figs. 2 and 3).

HY5 is a key regulator of light-induced anthocyanin biosynthesis in various plant species, and it acts downstream of multiple photoreceptors, such as PHY, CRY, and UVR8, in the signal transduction pathways^37^. In the presence of light, the activated photoreceptors inhibit COP1/SPA activity by distinct regulatory mechanisms, leading to the stabilization of HY5^38^. HY5 activates the expression of anthocyanin pathway genes, such as *CHS, DFR*, and *LDOX*, as well as regulatory genes (such as *MYBs*) by directly binding to the G-box or ACE (ACGT-rich elements) motifs in the promoters of the target genes. In Arabidopsis, HY5 regulates anthocyanin biosynthesis directly by binding to the promoters of pathway genes and indirectly by activating the expression of PAP1 (MYB75)^39^ and the MYB-like domain TF MYBD^40^. HY5 binds to the *MYBD* and *PAP1* promoter to induce expression in response to light. *MYBD* or *PAP1* overexpression induces the expression of anthocyanin pathway genes such as *DFR* and *ANS*. MYBD represses the activity of MYBL2, a negative regulator of anthocyanin biosynthesis, resulting in the promotion of anthocyanin biosynthesis^40^ (Fig. 2b). Similar to Arabidopsis, in apple (*Malus domestica*), MdHY5 stimulates anthocyanin biosynthesis by inducing the expression of *MdMYB10* and *MdMYBDL1*, and HY5 directly bind to the G-box motifs in these *MYB* promoters^41,42^. In apple, MdMYB1/10 are PAP1-like MYBs and components of the apple MBW complex, and MYBDL1 is closely related to Arabidopsis MYBD. MdHY5 and MdMYBDL1 inhibit the expression of *MdMYB16* and *MdMYB308*, which are repressors of anthocyanin accumulation. Additionally, MdMYB308 interacts with MdbHLH3 and MdbHLH33, components of the apple MBW complex. MdMYB308-MdbHLH3/33 interaction likely affects MBW complex formation and influences anthocyanin accumulation in response to light^42^ (Fig. 2c). Exposure to UV-B induces anthocyanin accumulation in apple. Recent studies identified two UV-B responsive WRKY TFs, MdWRKY71-L and MdWRKY72, that promote anthocyanin accumulation in apple by inducing the expression of *MdHY5* and *MdMYB1*^43,44^. In blood orange (*Citrus* x *sinensis*), HY5 regulates the expression of the MYB TF *Ruby* to promote anthocyanin accumulation^45^. In peach (*Prunus persica*), PpHY5 regulates the expression of biosynthetic genes, such as *PpCHS1, PpCHS2, PpDFR1*, and regulatory gene *PpMYB10.1* to induce anthocyanin accumulation^46^. In red-skinned pears (*Pyrus pyrifolia*), PyHY5 directly binds to the G-box element in the promoters of *PyMYB10* and *PyWD40*, thereby enhancing their expression and resulting in increased anthocyanin accumulation^47^. SlHY5 regulates blue-light-induced anthocyanin accumulation by directly binding to the CHS and DFR promoters in tomato^37^.

In Arabidopsis, PIF3 positively, while PIF4 and PIF5 negatively, regulate anthocyanin biosynthesis in response to different light conditions (Fig. 2b). HY5 acts synergistically or antagonistically with PIFs to regulate different biological processes. Overexpression of *PIF3* (*PIF3*-OE) increases anthocyanin levels under far-red light, while *pif3* mutants exhibit reduced anthocyanin accumulation. However, the anthocyanin level in the *hy5* mutant overexpressing *PIF3* (*PIF3-OEhy5*) is similar to that in the *hy5* single mutant, suggesting that the positive effects of PIF3 require a functional HY5. Indeed, PIF3 and HY5 activate the same set of genes in the anthocyanin biosynthetic pathway, and PIF3-mediated activation is dependent on HY5. However, PIF3 and HY5 do not compete for the same binding motif. PIF3 binds to the G/E-box, while HY5 binds to ACE motifs in the promoters of anthocyanin pathway genes^11^. In Arabidopsis, red light induces anthocyanin accumulation, which is enhanced in *pif4* and *pif5* mutants but suppressed in *PIF4*-OE and *PIF5*-OE plants. Consistent with anthocyanin phenotype, the expression of genes encoding anthocyanin pathway enzymes and regulators, such as *CHS*, *F3*′*H*, *DFR*, *LDOX*, *PAP1*, and *TT8*, is increased in *pif4* and *pif5* mutants but decreased in *PIF4* and *PIF5*-OE plants. Additionally, PIF4 and PIF5 repress red light-induced promoter activities of *DFR* and *F3’H*, and PIF5 binds to the G-box motif in the *DFR* promoter to repress its expression. These findings collectively suggest that PIF4 and PIF5 act as negative regulators of red light-induced anthocyanin accumulation^48^. A recent study demonstrated that PIF4 binds to the G-box motif in the *PAP1* promoter to repress its expression and negatively regulates anthocyanin biosynthesis in response to white light^49^. Anthocyanin accumulation is also suppressed in response to other abiotic factors such as salt and phytohormone BA (6-benzylaminopurine). It has been demonstrated that PIF4 interacts with the R2R3 MYB TF PAP1 and competes with the bHLH TF TT8 for binding to PAP1, thus inhibiting the formation of the MBW complex. This mechanism is likely involved in PIF4 and PIF5 inhibition of anthocyanin accumulation in red light^50^. The function of HY5 in PIF4/5-mediated anthocyanin biosynthesis is not explored in these studies. PIFs also negatively regulate anthocyanin accumulation in other plants such as apple and pear. PIF8 positively regulates anthocyanin biosynthesis in pear skin and calli by activating the expression of *CHS*^51^. In apple, MdPIF7 is a negative regulator, while MdHY5 and MdBBX23 are positive regulators of anthocyanin biosynthesis. *MdHY5* is regulated by MdBBX23. MdPIF7 interacts with MdBBX23 and attenuates the transcriptional activation of MdBBX23 on *MdHY5*, thereby repressing anthocyanin accumulation. In apple MdbZIP44 and MdERF38 interact with MdMYB1 to regulate anthocyanin biosynthesis. MdPIF7 interacts with MdERF38 and MdbZIP44 and weakens the formation of the MdERF38/MdbZIP44-MdMYB1 complex to affect anthocyanin accumulation (Fig. 2c)^52^.

The B-box (BBX) proteins regulate anthocyanin biosynthesis in response to light either independently or by interacting with HY5^53,54^. In Arabidopsis, anthocyanin biosynthesis is positively regulated by BBX21/22/23^18,55^ but generally negatively regulated by BBX24/25^56^ (Fig. 2b). In apple (*Malus domestica*), while a group of light-inducible BBX proteins (BBX1/20/21/22/33) promote anthocyanin biosynthesis by regulating the expression of *HY5* and *MYB1/10*^57,58^, MdBBX37 inhibits anthocyanin biosynthesis through two different mechanisms (Fig. 2c). MdBBX37 interacts with MdMYB1 and MdMYB9, preventing them from binding to the promoters of the anthocyanin pathway genes. Alternatively, MdBBX37 binds to the *MdHY5* promoter to repress its expression^59^. Similar to apple, a number of BBX proteins regulate anthocyanin accumulation in pear (*Pyrus pyrifolia*). PpBBX16 and PpBBX18 interact with PpHY5 to activate the expression of *PpMYB10* and anthocyanin pathway genes to regulate anthocyanin accumulation. PpBBX21, on the other hand, acts as a negative regulator by inhibiting the formation of the PpBBX18–PpHY5 complex, thus repressing anthocyanin biosynthesis^60,61^. In strawberry (*Fragaria* x *ananassa*), FaBBX22 interacts with FaHY5 to enhance anthocyanin accumulation, and the interaction upregulates the expression of anthocyanin biosynthetic genes such as *FaPAL, FaANS, FaF30H*, and *FaUFGT1*, as well as the transporter *FaRAP*, in a light-dependent manner^62^. In summary, light-induced anthocyanin biosynthesis is regulated by a well-conserved group of TFs, i.e. PIFs, HY5, BBX, and MYBs in Arabidopsis and their homologs in other plant species, such as apple, pear, and strawberry. In addition, other regulators, such as WRKYs and the AP2/ERFs, have been identified in apple as the regulators of anthocyanin biosynthesis.

MicroRNAs (miRNAs) are a class of endogenous small RNAs, typically 21 or 22 nucleotides in length, found in both animals and plants^63^. They are important mediators of post-transcriptional gene regulation that fine-tune many developmental and metabolic processes in plants. MiRNAs are known to regulate biosynthesis of SM, including anthocyanins in Arabidopsis and other plant species^28,29,64–66^, terpenoid indole alkaloids (TIAs) in *Catharanthus roseus*^26^, and sesquiterpenes in Arabidopsis^67^. In this context, we will specifically discuss the influence of light on miRNA regulation of anthocyanin biosynthesis in plants.

In Arabidopsis, the expression of miR858a, a positive regulator of anthocyanin biosynthesis, is induced by light. Light-induced expression of miR858a is HY5-dependent, and HY5 binds to the miR858a promoter to regulate its expression. Overexpression of miR858a partially complements the long hypocotyl phenotype of the *hy5* mutant and rescues the anthocyanin levels in the mutant. Additionally, overexpression of miR858a in wild-type Arabidopsis significantly induces expression anthocyanin pathway genes, such as *DFR*, *ANS*, *TT8*, *GL3*, and increases anthocyanin accumulation^68^. miR858a and HY5 regulate the activity of MYBL2, a negative regulator of anthocyanin, by translational inhibition and chromatin modification. Overall, multiple regulatory mechanisms involving miRNA858a and HY5 regulate the light-induced anthocyanin accumulation in Arabidopsis. In apple, light induces anthocyanin accumulation but reduces lignin level. A recent study has demonstrated that the inverse correlation between anthocyanin and lignin accumulation is mediated by the light-induced miRNA7125^69^. RNAseq and miRNAseq analyses, combined with experimental data, suggest that cinnamoyl-coenzyme A reductase gene (*CCR*), a key enzyme in the lignin pathway, is the target of miRNA7125. Additionally, both the activator MdMYB1 and the repressor MdMYB16 of anthocyanin biosynthesis bind to the miRNA7125 promoter to regulate its expression. The interplay among MdMYB1/MYB16, CCR, and miRNA7125 maintains the homeostasis between anthocyanin and lignin in response to light^69^. In red Chinese sand pear (Pyrus spp.), light-induced anthocyanin accumulation is regulated by the PyPIF5-*PymiR156a*-PySPL9-PyMYB114/MYB10 module. PyMYB114 and PyMYB10 are components of the MBW complex that positively regulates anthocyanin biosynthesis. In contrast, pySPL9 and PyPIF5 negatively regulate anthocyanin biosynthesis. PySPL9 is a target of PymiR156, and it interacts with PyMYB114/MYB10, inhibiting the MBW complex formation and affecting anthocyanin accumulation. PyPIF5 regulates the PymiR156 expression by binding to the G-box motif in the promoter. Light induces the expression of PymiR156 but reduces that of *PyPIF5*. The light-induced upregulation of miRNA156 results in the degradation of *PySPL9*, leading to higher anthocyanin accumulation. In addition, overexpression of miR156 induces anthocyanin accumulation in pear fruit skin. Collectively, these studies underscore the importance of miRNAs in light-induced anthocyanin accumulation (Fig. 3a)^70^.

Light plays a crucial role in plant adaptation to changing environments and is perceived by photoreceptors that transduce different wavelengths of light into physiological responses through intracellular signaling pathways^71^. The protein kinases (PKs) are major components of various signaling pathways including light. PKs regulate many biological processes in plants, including growth and development, phytohormone signaling, and responses to abiotic and biotic factors. PKs are activated in response to various signals and phosphorylate diverse substrates, including TFs and enzymatic proteins, to induce appropriate responses. In Arabidopsis, PAP1 is a component of the MBW complex that positively regulates anthocyanin biosynthesis. The mitogen-activated protein kinase 4 (MAPK4) interacts with PAP1 to increase its stability, which is essential for anthocyanin accumulation in response to light^72^. In apple, MPK4 has been reported to mediate the phosphorylation of MYB1 to enhance light-induced anthocyanin accumulation^73^. Analysis of apple fruit phosphoproteome in response to light identified HY5 to be differentially phosphorylated. Protein-protein interaction and phosphorylation assays revealed that MPK6 phosphorylates HY5 and its activity is increased by light, which is crucial for increased anthocyanin accumulation^74^. Apart from MPKs, other groups of kinases also regulate anthocyanin biosynthesis. In Arabidopsis, the Sucrose non-fermenting Related Kinase 1 (SnRK1) represses anthocyanin biosynthesis at both transcriptional and post-translational levels. SnRK1 inhibits the expression of *PAP1*, triggering the dissociation of the MBW complex and the degradation of PAP1 (Fig. 3b)^75^.

In Arabidopsis, anthocyanin accumulation is induced by light and suppressed in the dark. COP1 and SPA are components of the E3 ubiquitin ligase complex, and are involved in the dark suppression of anthocyanin accumulation. E3 ubiquitin ligases are components of the 26 S proteasomal degradation machinery. The *cop1* mutant accumulates anthocyanin in the dark when a functional PAP1 is present. Protein-protein interaction studies indicated that PAP1 and PAP2 interact with COP1 and SPA1, and PAP1 and PAP2 proteins are degraded in the dark but stable under light. In addition, PAP2 degradation is dependent on the 26 S proteasome and COP1^76^. Similarly, in apple, MdMYB1, a homolog of PAP1 and positive regulator of anthocyanin, is accumulated under light but degraded in the dark. MdCOPs interact with MdMYB1 and are necessary for the MYB1 degradation in the dark^77^.

### Regulation of stilbene biosynthesis by light

Stilbenes, particularly *trans*-Resveratrol (t-Res), are phenolic compounds found in various plant species such as grapes (*Vitis vinifera*). t-Res has gained significant attention due to its roles in plant defense mechanisms and its potential health-promoting properties. Stilbenes are derived from the general phenylpropanoid pathway, and stilbene synthase (*STS*) catalyzes the conversion of three units of malonyl-CoA and one unit of *p*-coumaroyl CoA into t-Res in a single step. Res accumulation is induced by UV light (UV-B and UV-C). UV-C is more effective than UV-B in inducing stilbenes, including Res, in grapes. The regulation of Res in response to UV has been extensively studied in grape vines. R2R3 MYB (VvMYB14 and VvMYB30) and WRKY (WRKY8) TFs are involved in the regulation of Res biosynthesis in response to UV irradiation. VvMYB14 and WRKY8 are activators, whereas MYB30 is a repressor of Res biosynthesis. UV-B and UV-C irradiation induce the expression of the grape *STS15/21, VvMYB14*, and *VvWRKY8* genes, but repress *VvMYB30*, suggesting that an activator-repressor module controls the accumulation of Res in response to UV^78^.

### Regulation of carotenoid and artemisinin biosynthesis by light

Terpenoids are synthesized from the five-carbon precursors isopentenyl pyrophosphate (IPP) and dimethylallyl pyrophosphate (DMAPP)^79^. They are classified based on the number of five-carbon units in their skeletons, ranging from hemi- (C5) to tetra- (C40) terpenoids. Terpene synthase (TPS) is the key enzyme responsible for synthesizing the basic backbone structures. In nature, terpenoids serve various biological functions^80^, for instance, carotenoids function as photosynthetic pigments, while certain terpenoids emit volatile floral scents to attract the pollinators. Many terpenoids are defense molecules against pathogens. In addition, various terpenoids possess therapeutic properties and have been used in the treatment of various diseases. For example, artemisinin, produced by *Artemisia annua* (sweet wormwood), is a well-known effective antimalarial drug. Light plays a crucial role in regulating biosynthesis and accumulation of terpenoids in many plant species^81–84^. Here, we highlight the roles of light on in regulation of the biosynthesis of two terpenoids, carotenoids and artemisinin.

**Fig. 4:**
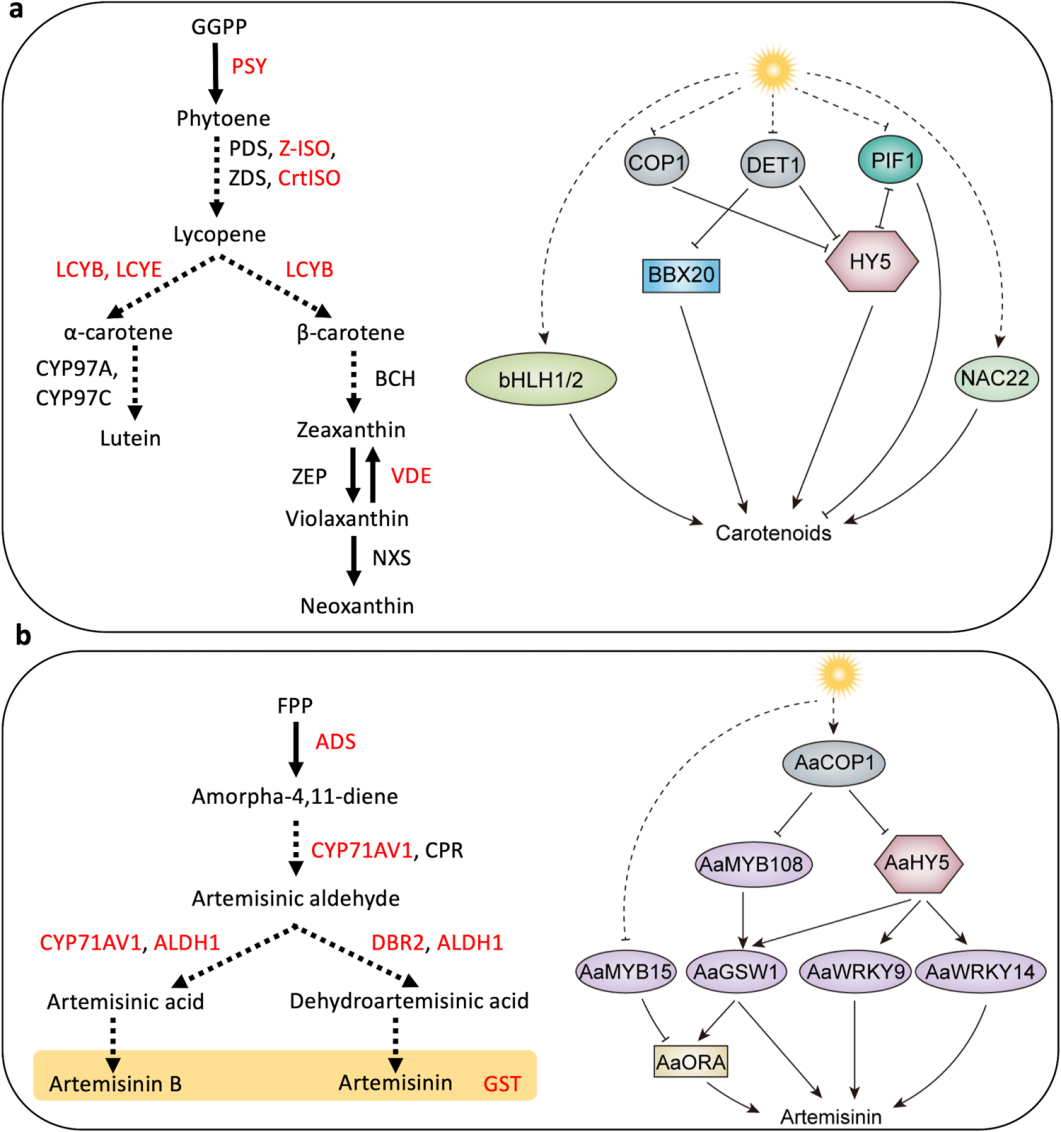
Light regulation of carotenoid and artemisinin biosynthesis. **a** Left panel, schematic diagram of the carotenoid biosynthetic pathway in pants. The light-responsive genes encoding carotenoid biosynthetic pathway enzymes are highlighted red. PSY phytoene synthase, PDS phytoene desaturase, Z-ISO ζ-carotene isomerase, ZDS ζ-carotene desaturase, CrtISO carotenoid isomerase, LCYB lycopene β-cyclase, LCYE lycopene ε-cyclase, CYP97A cytochrome P450 β-carotene hydroxylase, CYP97C cytochrome P450 ε-carotene hydroxylase, BCH β-carotene hydrolase, ZEP zeaxanthin epoxidase, VDE violaxanthin de-epoxidase, NXS neoxanthin synthase. Right panel, carotenoid biosynthesis is induced by light through the activators HY5, BBX20, bHLH1/2, and NAC22, as well as the repressors COP1, DET1, and PIF1. Light enhances carotenoid production by simultaneously inducing transcriptional activators bHLH1/2 and NAC22 while suppressing negative regulators COP1, DET1, and PIF1. HY5 and PIF1 inhibit each other and compete to regulate the carotenoid biosynthetic genes. Light suppresses DET1, which is a negative regulator of BBX20 and HY5. **b** Left panel, schematic diagram of the artemisinin biosynthetic pathway in *Artemisia annua*. The light-responsive genes encoding artemisinin biosynthetic pathway enzymes are highlighted in red. ADS amorpha-4,11-diene synthase, CYP71AV1 amorpha-4,11-diene hydroxylase (cytochrome P450 monooxygenase), CPR cytochrome P450 reductase, ALDH1 aldehyde dehydrogenase 1, DBR2 artemisinic aldehyde Δ11(13) reductase, GST glandular secretory trichome. Right panel, artemisinin biosynthesis is induced by light through the activators AaHY5, AaMYB108, three WRKYs, and AaORA, as well as the repressors AaCOP1 and AaMYB15. AaHY5 and AaMYB108 act upstream of the WRKYs and AaORA to regulate their activities. AaHY5 and AaMYB108 are targeted by AaCOP1 for degradation in the dark. The expression of *AaMYB15*, a repressor of artemisinin biosynthesis, is repressed by light. Solid and dotted arrows indicate direct and indirect regulation, respectively. AaORA *Artemisia annua* OCTADECANOID-DERIVATIVE RESPONSIVE AP2-DOMAIN PROTEIN, AaGSW1 GLADULAR TRICHOME SPECIFIC WRKY1. Solid and dotted T-bars indicate direct and indirect repression, respectively.

Carotenoids are tetra-terpenoids widely distributed in plants and are responsible for the red, orange, or yellow colors observed in flowers, fruits, and roots^85^ (Fig. 1). In addition to their roles in pigmentation, carotenoids serve as essential photosynthetic pigments in chloroplasts. The primary function of carotenoid is to protect the photosynthetic apparatus from photooxidative damage caused by excessive illumination^85,86^. The biosynthesis of carotenoids is intricately regulated by light and is closely associated with the development of chloroplasts^87,88^. Carotenoids biosynthesis starts with the formation of phytoene, which is produced from two molecules of geranylgeranyl pyrophosphate (GGPP) catalyzed by phytoene synthase (PSY)^85^. Phytoene undergoes a series of enzymatic steps, including desaturation, cyclization, and hydroxylation, resulting in the production of carotenoids with a wide range of colors (Fig. 4). Among the biosynthetic enzymes involved in carotenoid biosynthesis, PSY is considered a rate-limiting enzyme and its gene expression is regulated by light. Recent evidence suggests that the major light signaling factors, including PIFs, HY5, BBXs, COP1, and DET1, regulate the expression of genes involved in carotenoid biosynthesis and accumulation^88^. PIFs play a negative regulatory role in carotenoid biosynthesis by repressing the expression of *PSY*. The PIF-PSY regulatory module has been observed in many plant species, including Arabidopsis^89^, tomato^90^, and grape berry^91^. PIFs directly repress *PSY* in Arabidopsis and tomato fruit. In contrast, HY5 positively regulates carotenoid biosynthesis in both Arabidopsis^12^ and tomato^92,93^. In tomato fruit, HY5 directly regulates multiple carotenoid biosynthetic genes, including *PSY*, *Z-ISO* (*15-cis-zeta-carotene isomerase*), *CrtISO1* (*carotene isomerase 1*), *LCYB* (*lycopene beta-cyclase*), *LCYE* (*lycopene epsilon-cyclase*), and *VDE* (*violaxanthin de-epoxidase*), indicating a broader regulatory role for HY5 on the carotenoid pathway compared to PIFs. Both PIF1 and HY5 directly bind to the G-box in the *PSY* promoter in Arabidopsis, and these two proteins exhibit antagonistic effects on *PSY* expression, highlighting their opposing roles in carotenoid biosynthesis^12^. BBX proteins have also been implicated as regulators of carotenoid biosynthesis in tomato^19^ and pepper^94^. However, their regulatory roles appear to be limited to specific carotenoid biosynthetic genes, such as *PSY* in tomato and *CCS* (*capsanthin-capsorubin synthase*) in pepper. Silencing of *COP1* or *DET1* significantly enhanced the accumulation of carotenoids in tomato^92,95^, suggesting the negative roles of both light-regulated E3 ubiquitin ligases in carotenoid metabolism (Fig. 4a).

The regulation of carotenoids in response to light involves not only the core light signaling regulators, but also other TFs that play important roles in various plant species. In papaya (*Carica papaya*), two bHLH TFs, *CpbHLH1* and *CpbHLH2*, are induced by light during fruit ripening and activate carotenoid biosynthetic genes^96^. Similarly, during kumquat (*Fortunella crassifolia*) fruit ripening, a NAC (NAM/ATAF/CUC2) family TF gene, *FcrNAC22*, is upregulated by light, triggering the expression of carotenoids pathway genes^97^. However, the relationships of these bHLH and NAC TFs with the core light signaling components need further investigation and verification.

Artemisinin is an oxygenated sesquiterpene synthesized and stored in the glandular secretory trichomes (GSTs) of *A. annua* leaves^98^ (Fig. 1). The precursor of artemisinin, amorpha-4,11-diene, is formed by the cyclization of farnesyl diphosphate (FPP). Subsequently, amorpha-4,11-diene is converted to artemisinin through several enzymatic steps and non-enzymatic reactions (Fig. 4). Light induces artemisinin accumulation through upregulating key pathway genes, such as *CYP71AV1* and *AaDBR2* (*artemisinic aldehyde Δ11(13) reductase*). The biosynthesis of artemisinin is positively regulated by AaHY5^99^. AaHY5 directly induces the expression of three WRKY TFs, *AaGSW1* (Glandular trichome specific WRKY1)^99^, *AaWRKY9*^100^ and *AaWRKY14*^101^. These WRKY TFs, in turn, activate genes encoding key enzymes in the artemisinin biosynthetic pathway. AaGSW1 and AaWRKY14 directly target *CYP71AV1*^98,101^ while AaWRKY9 directly activates *AaDBR2* and *AaGSW1*^100^. In addition, AaGSW1 indirectly induces artemisinin pathway enzymes through activating the expression *AaORA* (*octadecanoid-derivative responsive AP2-domain protein*)^98^, another transcriptional activator of artemisinin biosynthesis^102^. A recent study has shown that AaGSW1 interacts with the R2R3 MYB TF AaMYB108 to induce the expression of *CYP71AV1*, which encodes a cytochrome P450 monooxygenase in the artemisinin pathway^103^. Moreover, both AaHY5 and AaMYB108 are targets of AaCOP1^103^, further highlighting the complexity of the transcriptional regulatory network controlling artemisinin biosynthesis in response to light (Fig. 4b).

The development of GSTs in *A. annua* leaves is crucial for the accumulation of artemisinin. Recent research has shed light on the regulatory mechanisms governing GST development and initiation, particularly involving the AaCOP1 protein^104^. In the darkness, AaCOP1 interacts with and facilitates the degradation of AaSEP1, a positive regulator of GST initiation. The AaSEP1-AaMYB16-AaHD1 regulatory complex regulates GST initiation^104,105^. AaHD1 directly activates the expression of *AaGSW2*, an activator of GST initiation^106^. AaMYB16 enhances the activation of AaHD1 on the *AaGSW2* promoter through their interaction, and the interaction between AaSEP1 and AaMYB16 further strengthens this activation process. AaCOP1 not only regulates the protein level of AaSEP1 but also influences the transcript level of AaSEP1 in response to light. The specific mechanism through which light induces the transcription of AaSEP1 remains unknown^104^.

### Regulation of the biosynthesis of alkaloids and nitrogen-containing metabolites by light

Alkaloids, which are nitrogen-containing compounds, have been studied extensively for their functions in plant defense against pathogens and insects. Different types of alkaloids and nitrogen-containing metabolites, such as terpenoid indole alkaloids (TIA) in *Catharanthus roseus* (Madagascar periwinkle)^107^, steroidal glycoalkaloids (SGA) in *Solanum lycopersicum* (tomato)^108–110^, and glucosinolates (GSL) in Arabidopsis^111^, have been the focus of research for their roles in plant defense and benefits in human health. While the biosynthesis and regulation of these SM by the phytohormone jasmonate acid (JA) have been a major focus of many studies^112^, recent research has highlighted the regulatory roles of light in the biosynthesis of GSLs^113^, alkaloids including vindoline in *C. roseus*^114^, camptothecin in *Camptotheca acuminata* (Chinese Happy Tree)^115^, and SGAs in tomato (Fig. 1)^116^.

**Fig. 5:**
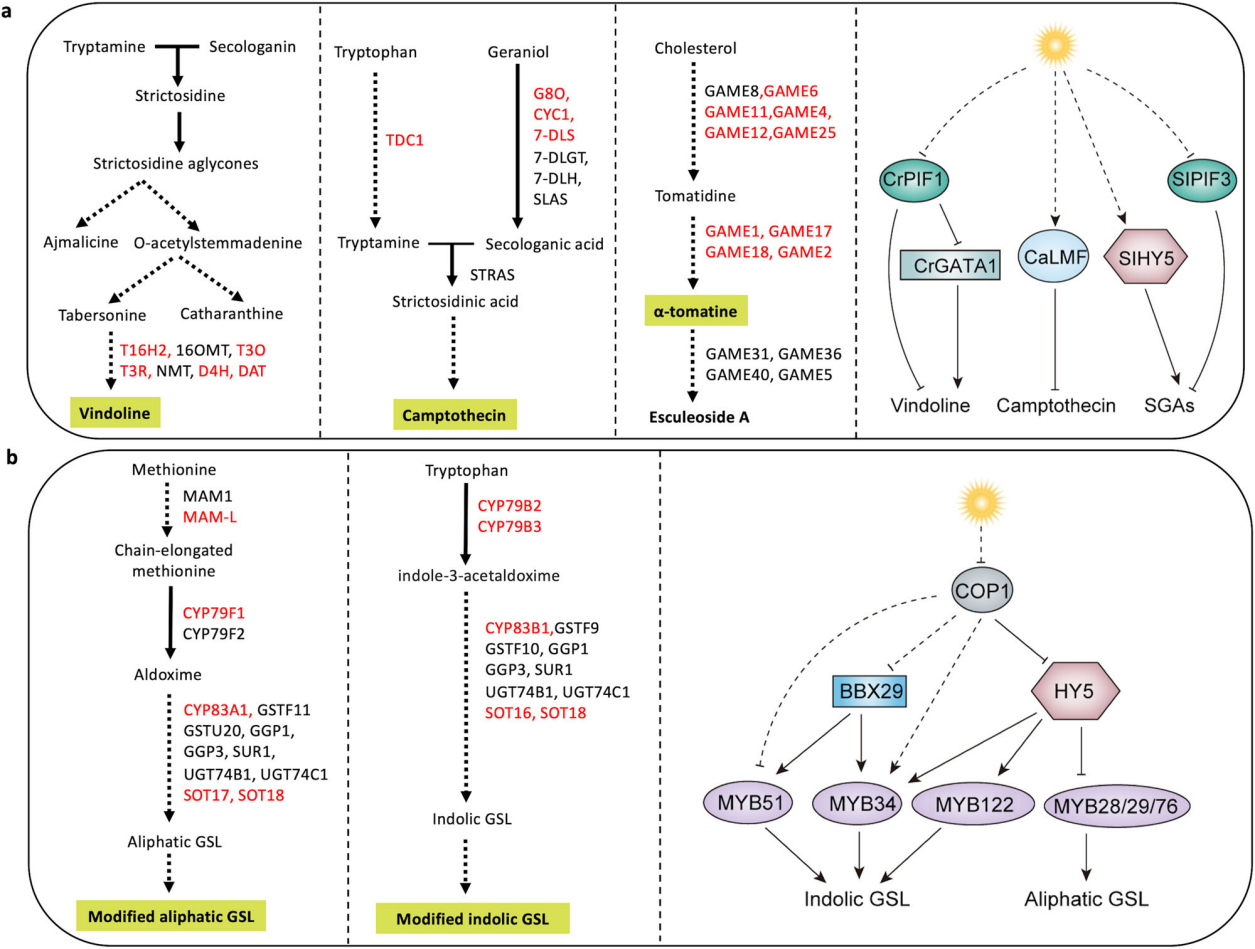
Light regulation of TIA, SGA, and GSL biosynthesis. **a** Left three panels, schematic diagrams of the vindoline, camptothecin, and steroidal glycoalkaloid (SGA) biosynthetic pathways in *Catharanthus roseus*, *Camptotheca acuminata*, and tomato (*Solanum lycopersicum*), respectively. The light-responsive genes encoding the enzymes are highlighted in red. T16H2 tabersonine 16-hydroxylase 2, 16OMT 16-O-methyl transferase, T3O tabersonine 3-oxygenase, T3R tabersonine 3-reductase, NMT N-methyltransferase, D4H desacetoxyvindoline-4-hydroxylase, DAT deacetylvindoline-4-Oacetyltransferase, G8O geraniol-8-oxidase, CYC1 CYCLASE1, 7-DLS 7-deoxyloganetic acid synthase, 7-DLGT 7-deoxyloganetic acid glucosyltransferase, 7-DLH 7-deoxyloganic acid hydroxylase, SLAS secologanic acid synthase, TDC1 tryptophan decarboxylase 1, STRAS strictosidinic acid synthase, GAME GLYCOALKALOID METABOLISM. Right panel, light-regulation of vindoline, camptothecin, and SGAs in respective plants. Light-induced TIA (vindoline) biosynthesis in *Cathranthus roseus* is regulated by transcription factors CrGATA1 and CrPIF1. The expression of *CrGATA1*, an activator of vindoline biosynthesis, is induced by light. CrPIF1 suppresses CrGATA1, but it is degraded by light, thus releasing CrGATA to activate the vindoline pathway. CrPIF1 also directly represses vindoline biosynthesis. Camptothecin biosynthesis in *Camptotheca acuminata* is repressed by light through activation of CaLMF. In tomato, light induces SGA biosynthesis by upregulating the activator SlHY5 while suppressing the repressor SlPIF3. **b** Left two panels, schematic diagram of the aliphatic and indolic glucosinolates (GSL) biosynthetic pathway in *Arabidopsis thaliana*. The light-responsive genes encoding the enzymes are highlighted in red. Methylthioalkylmalate synthase (MAM1, MAM3), cytochrome P450 monooxygenases (CYP79B2, CYP79B3, CYP79F1, CYP79F2, CYP83A1, CYP83B1), glutathione S-transferase (GSTF9, GSTF10, GSTF11, GSTU20), γ-glutamyl peptidase (GGP1, GGP3), SUR1 SUPERROOT 1, UDP-glycosyltransferase (UGT74B1, UGT74C1), sulfotransferases (SOT16, SOT17, SOT18). Right panel, the biosynthesis of indolic and aliphatic GSL in Arabidopsis is regulated by light through the activators MYB34/51/122 and MYB28/29/76. BBX29 is a positive regulator of MYB52/34. Light inhibits COP1 which is a repressor of HY5, and the activated HY5 positively regulates *MYB34/51/122*, thus increasing indolic GSL biosynthesis. However, HY5 represses MYB28/29/76, thus reducing aliphatic GSL biosynthesis. CaLMF, *Camptotheca acuminata* Light-Mediated Camptothecin Biosynthesis Factor. Solid and dotted arrows indicate direct and indirect regulation, respectively. Solid and dotted T-bars indicate direct and indirect repression, respectively.

*C. roseus* is the source of more than 130 TIAs including the anticancer drugs vincristine and vinblastine^117^. TIA biosynthesis is a highly complex process involving more than 30 enzymes and 20 regulatory proteins. The biosynthesis of TIAs starts with the condensation of tryptamine, derived from the indole branch of the pathway, and secologanin, derived from the iridoid branch, to form strictosidine, which is the universal precursor for all TIAs, including ajmalicine, serpentine, tabersonine, catharanthine, and vindoline (Fig. 5)^118,119^. Vincristine and vinblastine are derived from the condensation of vindoline and catharanthine. Vindoline is preferentially accumulated in the aerial parts of the plant and is induced by light in a phytochrome-dependent manner^120^. The biosynthesis of vindoline from tabersonine involves seven enzymatic steps^121^, and the expression of five of the seven genes, including *T16H2 (tabersonine 16-hydroxylase)*, *T3O (tabersonine 3- oxidase)*, *T3R (tabersonine 3-reductase)*, *D4H (desacetoxyvindoline-4-hydroxylase)*, and *DAT* (*deacetylvindoline 4-Oacetyltransferase*) is induced by light^114^. A recent study has revealed that the core components of the light signaling influence vindoline biosynthesis in *C. roseus*. CrPIF1 is a negative regulator of vindoline biosynthesis while the GATA-type zinc finger family TF, CrGATA1, acts as a positive regulator^114^. Specifically, CrPIF1 represses the expression of *CrGATA1*, *T16H2*, and *DAT*, whereas CrGATA1 activates the expression of the five vindoline biosynthetic genes that are responsive to light^114^. It has been suggested that the CrPIF1 suppresses vindoline biosynthesis in the dark, and the degradation of CrPIF1 upon exposure to light triggers the induction of vindoline biosynthesis by CrGATA1 (Fig. 5a)^114^.

Camptothecin is a TIA produced in *C. acuminata* and has shown antitumor activity against cancers in breast, ovarian, colon, lung, and stomach^122,123^. Camptothecin is accumulated in all tissues with the shoot apex and young leaf accumulating the highest levels^124^. In *C. acuminata*, camptothecin biosynthesis begins with the condensation of tryptamine and secologanic acid to produce strictosidinic acid (Fig. 5). The subsequent steps in camptothecin biosynthesis have not been fully elucidated^124^. Although it has been known for some time that camptothecin production is negatively regulated by light, the regulatory mechanism remained unknown^125^. A recent research has revealed that light suppresses camptothecin biosynthesis by affecting the expression of four crucial camptothecin biosynthesis genes, *CaTDC1*, *CaG8O*, *CaCYC1*, and *Ca7-DLS*^115^. A bZIP TF, CaLMF (Light-Mediated camptothecin Biosynthesis Factor), has been identified as a regulator of the light-induced suppression of camptothecin biosynthesis. Light induces the expression of *CaLMF*, which represses the expression of four camptothecin biosynthetic genes (Fig. 5a)^115^. Although CaLMF shares significant sequence similarity with AtHY5, its functional role as a HY5-like protein requires further investigation.

*Solanaceous* species, such as tomato and potato, produce SGAs, which are toxic alkaloids involved in defense against biotic stress. In tomato, the major SGA is α-tomatine, which is highly accumulated in immature fruits to provide protection against pathogens and predators^108^. SGAs are derived from cholesterol through a series of enzymatic reactions, including hydroxylation, oxidation, transamination, and glycosylation. The enzymes catalyzing these steps are well characterized and known as GLYCOALKALOID METABOLISM enzymes (GAMEs) (Fig. 5)^109,110^. SGA biosynthesis is light inducible as most of the *GAMEs* are upregulated in response to light exposure^126^. The core light signaling components, including the activator SlHY5^116,126^ and the repressor SlPIF3^126^, regulate SGA biosynthesis. Specifically, SlHY5 directly activates the genes encoding GAME1, GAME4, and GAME17, while SlPIF3 acts as a repressor (Fig. 5a)^126^. These findings suggest that the tomato SGAs biosynthesis is tightly regulated by the core light signaling components.

GSLs are a diverse group of nitrogen and sulpfur-containing SM found in the Brassicaceae family plants including Arabidopsis^111,127^. GSLs play important roles in plant defense against various pests and diseases and have significant medicinal values^127,128^. They can be converted by intestinal bacteria into isothiocyanates, which have anti-inflammatory, anti-cancer, anti-obesity, and neuroprotective properties^129^. Recent research has shed light on the molecular mechanisms that govern the regulation of two major types of GSLs, aliphatic and indolic GSL, which are derived from the amino acid methionine and tryptophan, respectively (Fig. 5). The effects of light on GSL biosynthesis have been studied in many plant species^113,130,131^. The indolic and aliphatic GSL biosynthetic pathways can be divided into three independent stages, i.e. chain elongation of the amino acids, construction of the GSL structure, and secondary modifications of the amino acid side chain^132^. Light upregulates six GSL biosynthetic genes, including three involved in the biosynthesis of aliphatic GSL (*CYP79F1*, *MAM-L*, and *SOT17*), two in indolic GSL (*CYP79B2* and *SOT16*), and one associated with both types of GSL (*SOT18*)^113^. The expression of GSL pathway genes is regulated by a group of MYB TFs in Arabidopsis. The biosynthesis of aliphatic GSL is positively regulated by MYB28, MYB29, and MYB76, while that of indolic GSL is regulated by MYB34, MYB51, and MYB122^133,134^. The *MYB* genes associated with aliphatic GSL are induced by light and repressed in dark, while the expression patterns of those associated with indolic GSL vary in response to light^113^. Expression of *MYB51* is repressed in the dark but not induced by light, whereas that of *MYB34* is induced by light but not affected in the dark. *MYB122* is induced in the dark but does not respond to light. Light regulation of GSL is partially dependent on HY5, which positively regulates *CYP79F1*, *CYP79B2*, and *SOT18*. Expression of all three aliphatic GSL-associated *MYB28*, *MYB29,* and *MYB76* are strongly induced by light in the *hy5* mutant compared to wild-type Arabidopsis, whereas that of indolic GSL-associated *MYB34* and *MYB122* are repressed, suggesting that HY5 may act as a repressor of aliphatic GSL and an activator of indolic GSL. Interestingly, although the aliphatic MYBs are strongly induced in the *hy5* mutant, the degree of upregulation of the pathway genes are not significant, and, therefore, the hierarchy of MYBs and HY5 still needs to be established^113^. In addition, the BBX family TF AtBBX29 activates GSL biosynthesis in response to light, possibly acting through MYB34 and MYB51^135^. COP1, the negative regulator of light signaling pathway, plays a positive role in regulating GLS biosynthesis^134^. In *cop1* mutant lines, the reduced accumulation of both types of GSLs is consistent with downregulation of the aliphatic GSL biosynthetic gene *CYP83A1* and the indolic GSL biosynthetic genes *CYP79B3* and *CYP83B1*, as well as *MYB34*. However, the expression of *MYB51* and *MYB122* is upregulated in *cop1* mutant lines. In contrast to the positive regulator HY5, some activators of GSL biosynthesis are negatively regulated by light, with COP1 playing a positive role in this regulation (Fig. 5b). These findings highlight the complexity of the regulatory network governing GSL biosynthesis in response to light.

In summary, alkaloids, such as TIAs in *C. roseus* and SGA in tomato, are regulated by well-conserved light signaling factors such as PIFs and HY5. PIFs negatively affect the biosynthesis of TIAs and SGA. HY5 is a positive regulator of SGA in tomato, while the HY5-like factor, CaLMF, is a negative regulator of camptothecin in *C. accuminata*. Whether HY5 or HY5-like factors are involved in the regulation of TIAs in *C. roseus* remains known. The biosynthesis of aliphatic and indolic GSL is differentially regulated by HY5. Furthermore, other factors, such as MYBs and GATA-type TFs, regulate TIA and GSL biosynthesis in plants in response to light.

### Conclusion and Future Perspective

The biosynthesis of SM is influenced by many environmental factors including light (Fig. 1). However, this process is energy-intensive, and excessive accumulation of certain SM can be toxic to cells. Consequently, plants have evolved intricate regulatory mechanisms to prevent overactivation and fine-tune SM biosynthesis. Accumulating evidence suggests that the regulations of most SM biosynthetic pathways are conserved across plant species. One example of such conservation is observed in the regulation of anthocyanin biosynthesis, which is controlled by the MBW complex, in a wide range of plants, including the model plant Arabidopsis. Another instance is the regulation of terpene biosynthesis, where the bHLH TF MYC2 or its orthologues govern the biosynthetic process from the eudicot Arabidopsis to the primitive land plant, *Marchantia polymorpha*^136^. Furthermore, this conserved nature extends to the biosynthesis of structurally diverse SM. For example, the biosynthesis of TIAs in *C. roseus*, SGA in tomato and potato, and nicotine in tobacco are regulated MYC2 and a group of phylogenetically related JA-responsive AP2/ERFs (Apetala 2/Ethylene Responsive Factors)^137–141^. The conserved nature of the regulatory mechanism is also apparent in the light signaling pathway, in which the core light signaling pathway TFs PIFs, HY5, and BBX regulate the biosynthesis of diverse groups of SM, including anthocyanins, terpenes, and TIAs in various plant species. Nonetheless, some regulatory factors seem to be species-specific, *e.g*., some TFs involved in anthocyanin biosynthesis are found in apples but not Arabidopsis (Fig. 2b, c), suggesting that some plant species have evolved unique responses to light. Light and JA are important regulators of many SM. However, the relationship between JA and light signaling pathways and their combined effects on the regulation of SM biosynthesis have been relatively less studied. A previous study has shown that JA-induced artemisinin biosynthesis is dependent on light. Subsequent transcriptomic analysis of JA and light-treated *A. annua* seedlings has identified several candidate TFs potentially involved in the regulation of artemisinin biosynthesis^142^. Recently, a light and JA-inducible WRKY TF, AaWRKY9 that acts downstream of AaHY5, has been identified as a positive regulator of artemisinin biosynthesis^100^. Similarly, AaMYB108, identified from light and JA-treated transcriptomes, is a positive regulator of artemisinin biosynthesis^103^. Future studies will likely identify factors involved in the light and JA-dependent regulation of other plant SM.

While recent years have seen significant progress in understanding photo-regulation of SM biosynthesis, some existing questions are still left unanswered. Compared to transcriptional regulation, light-mediated posttranscriptional and posttranslational regulation of SM are insufficiently studied. Identification and functional characterization of additional protein kinases and miRNAs related to SM biosynthesis will further advance our knowledge on regulatory mechanisms. Follow-up questions would thus concern why such multilayered regulatory mechanisms are necessary. Phosphorylation of the MBW components by different light-responsive kinases can lead to stabilization or disassociation of the complex (Fig. 3). How then do plants balance such countereffects by kinases in response to light? In addition, many TFs are not only regulated by phosphorylation but also de-phosphorylation by protein phosphatases (PPs). The involvement of phosphatases in the photo-regulation of SM biosynthesis is currently poorly investigated. Furthermore, literature on other posttranslational modifications, such as SUMOylation, related to photo-regulation of SM biosynthesis is limited.

Phosphorylation-dephosphorylation by PKs and PPs, respectively, is an ancient regulatory mechanism that influences many biological processes in plants and animals^143^. Although several PKs that phosphorylate PIFs have been identified, related information on PP is limited ^3,144^. HY5 is shown to be phosphorylated by SPA kinases^145^. The regulatory roles of these PKs and PPs have been studied in respect to plant growth development^144,146^; however, it remains to be explored whether the PKs and PPs are involved in light-regulated SM biosynthesis.

SUMOylation is the reversible conjugation of small ubiquitin-related modifier (SUMO) peptides to the target protein and has emerged as an important posttranslational regulation mechanism in plants and animals^147^. SUMOylation has been implicated in many biological processes such as growth, development, biotic and abiotic stress, as well as phytohormone signaling and flowering. In Arabidopsis, SUMOylation regulates the stability of the bHLH TF ICE1 (Inducer of CBF Expression) by blocking its degradation which is required for activating freezing tolerance^148^. The bHLH TF MYC2 is crucial for Arabidopsis seedling development in blue light. Blue light induces the MYC2 SUMOylation, which is essential for MYC2 to function under blue light. SUMOylation increases the MYC2 stability and enhances its DNA binding ability. As MYC2 is also a key component of JA signaling and a regulator of many JA-responsive SM biosynthesis, future studies should include the investigation of crosstalk between light signaling and hormones, as well as SUMOylation with regard to SM biosynthesis^149^. In Arabidopsis, SUMOylation by the SUMO E3 ligase SIZ1 (SAP and MIZ1 domain containing ligase 1) increases PAP1 stability and is required for light-mediated anthocyanin accumulation^150^. It is reasonable to expect light-activated SUMOylation also affects other regulatory proteins; however, experimental verifications will be necessary.

Light affects the expression of approximately 20 percent of genes in the Arabidopsis and rice genomes^151^. The massive change in the transcriptomic landscape indicates that this process is modulated at chromatin level^152^. Plant epigenome is indeed dynamic, and extensive research reveals the effects of light-induced epigenetic changes, such as histone methylation, histone acetylation/deacetylation, and incorporation of histone variants, on plant growth and development. The core components of the light signaling pathway, such as HY5 and PIFs, interact with and recruit epigenetic factors to regulate gene expression^153^. In addition to epigenome, epitranscriptome (posttranscriptional modification of RNA such as methylation) is considered as a part of the epigenetic system and affects many aspects of plant development^153,154^. However, information is limited regarding the light and epigenetic regulation of SM biosynthesis in plants. In Arabidopsis, MYBD overexpression affects the acetylation status of the *MYBL2* promoter and represses *MYBL2* expression to promote anthocyanin accumulation^40^. Light-induced epigenetic changes affecting the biosynthesis of other SM, and the chromatin factors involved in this process remain to be characterized. The advances in omics technology will offer promising information on epigenetic regulation of SM biosynthesis. Recently, scATACseq (single-cell Assay for Transposase-Accessible Chromatin by sequencing) has emerged as a powerful technique to study epigenomic landscape of plants at a single-cell resolution in response to biotic or abiotic factors. Single-cell RNAseq has provided valuable insights into the spatial organization and the identification of new transporters in *C. roseus* TIA pathway^119,155^. A well-assembled and annotated genome is fundamental to the success of these approaches. Although challenging, integrating scATACseq and scRNAseq in response to light or other factors will yield significant information on the regulation of SM pathways in plants.
